# Use of Steel Slag as an Alternative to Aggregate and Filler in Road Pavements

**DOI:** 10.3390/ma14020345

**Published:** 2021-01-12

**Authors:** Giulio Dondi, Francesco Mazzotta, Claudio Lantieri, Federico Cuppi, Valeria Vignali, Celestino Sangiovanni

**Affiliations:** 1 Department of Civil, Chemical, Environmental and Material Engineering, University of Bologna, 40100 Bologna, Italy; giulio.dondi@unibo.it (G.D.); francescomazzotta86@gmail.com (F.M.); federico.cuppi3@unibo.it (F.C.); valeria.vignali@unibo.it (V.V.); 2 F.I.S. Impianti Interrati S.R.L., Grumello del Monte, 24064 Bergamo, Italy; c.sangiovanni@fisimpianti.eu

**Keywords:** recycled materials, steel slag, waste, construction and demolition waste

## Abstract

Today the use of Construction and Demolition Materials (CDM) can be considered as a suitable solution for the construction or the rehabilitation of road pavements. In this context, it is central to minimizing waste production, favoring the reuse through new production cycles to replace virgin natural raw materials. As illustrated in this study, steel slag has mechanical properties that justify its use as aggregate in the manufacture of bituminous mixes. In road construction, their use is focused on the substitution of fine aggregate and filler in bituminous mixtures. Mechanical characterizations, Marshall stability and indirect tensile resilient modulus (ITSM) tests were used to evaluate the laboratory performance of the mixtures. The research aims are to provide the use of these materials for the construction of the entire road pavement structure; in this study authors used these materials both in the characterization of cementitious layers and in those with bituminous conglomerate. In both cases, the use of steel slag has favored an increase of stiffness in the mixtures.

## 1. Introduction

The growing demand for raw materials has focused attention in the field of civil engineering toward the use of constructive modalities with alternative materials. The infrastructure sector has started to invest research resources on waste materials in order to reuse them in the construction of roads. Materials coming from the demolition of existing structures are a valuable resource, as they limit the use of raw materials reducing environmental impact.

The construction of road pavements requires a high amount of energy and non-renewable materials. For this reason, over recent decades, the use of recycled and/or reused materials is increased to develop sustainable products in this field.

The most commonly used recycling materials are derived from the demolition and steel industry waste, which can be reused in different percentages as aggregates in asphalt mixtures (RAP, steel slag, ceramics) in place of the virgin mineral aggregates [1].

In the literature, several studies have analyzed how to use these materials: while the objectives of reclaimed asphalt pavement (RAP) incorporation in asphalt mixtures are reusing both aggregate and binder [2], the waste powders such as waste brick [3,4], waste bleaching clay [5,6], rice husk ash [5], crumb rubber [7], and glass powder [8,9] were investigated to substitute the traditional limestone filler.

Among the material studied, steel furnace slag has been widely used and is accepted as a premium asphalt aggregate [9]. Slag of iron and steel used as aggregates for asphalt roads can be recovered as by-products. The steel slag is a residue of the production process of steel. The slag is mainly composed by: silica, alumina, titanium, iron sand and combinations of calcium and magnesium oxides. The function of the slag during the steelmaking process is to maintain the temperature of the molten iron. They can be classified into basic oxygen furnace slag (BOF), electric arc furnace slag (EAF) and ladle furnace slag (LF) [10,11]. In addition, Huaiwei wrote a review of several methods for treating the various stainless-steel wastes [12].

In the last decades, steel slag has been analyzed in several studies: basic oxygen furnace (BOF) slag was integrated into a warm [13,14], porous [15] hot mix asphalt [16], stone mastic asphalt (SMA-BOF), dense-graded asphalt concrete (DGAC-BOF) [17] or used as the main constituent in hydraulic road binder [18]. The results showed that the addition of steel slag into asphalt mixtures had high resistance to permanent deformation and moisture-induced damage. Steel slag included angular and rough-textured particles that would enhance the interlocking mechanism and provide good mechanical properties.

Electric arc furnace (EAF) steel slags, mostly produced in Italy, are most often used in concrete mixture [19] for building and construction purposes, and there are also studies related to the use of these slags in road pavements [20]. In fact, the literature reports studies on mechanical characteristics [21], durability [22], environmental characterization [23]. It has been estimated that with particular characteristics it is possible to partially replace natural aggregates with EAF granulated slag at a level of 15% [24]. Skaf et al. [25] studied all the possible reuse methods in a brief review. He recommends the use of EAF slag as a partial replacement of coarse aggregate [26], in all types of bituminous mixtures, after a suitable pretreatment. In this way, this improves durability, mechanical performance and sustainability of the mixtures.

Finally ladle furnace (LF) slag (white)**,** obtained by processing by refining in either a ladle furnace (LF) or specific converters for high-alloy steel, can be used to improve soil stabilization [27], or for the immediate use in masonry mortars and paving mixes for rural roads with low levels of traffic as a concreate mixture [28,29] or in asphalt mixes work properly as filler [30].

The object of the experimentation is the characterization of materials from treatment and recovery for use in the road pavement including foundation material and bituminous conglomerate.

**Figure 1:**
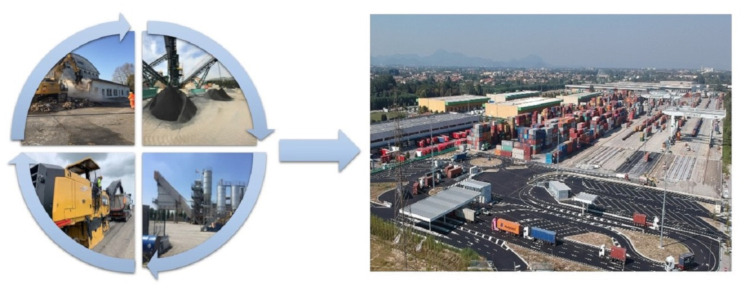
Material recovery cycle from construction and demolition materials (CDM) for the new Terminal of the Padua Interport.

These materials come from the recovery of waste from both the construction and the steel sector. The authors were able to test the use of recycled materials in place of virgin natural ones, for the construction of some infrastructures, including for example the new access yard and upgrading of the railway infrastructure of the new large terminal of the Padua Interport, built by Consital (BO). These concepts are illustrated in Figure 1 where the phases of reuse of the material from demolition to pavement construction are reported. This process is in line with Sustainable Development Goals and the targets set by European Commission to empower circular economy and to reduce CO_2_ emissions [31].

In particular, it was possible to achieve the objective of lower consumption of the soil and natural resources, after treatment and recovery, through production cycles in compliance with the technical standards of the sector UNI EN 13242, 13285, 12620, 13043 and the Circular of the Ministry of the Environment and Land Protection 15/07/2005 n. UL/2005/5205. The materials treated are listed below according to the classification CER (European Waste Catalog) listed below:

(1) Materials deriving from the recovery cycle of CDM (CER 17.01.01-17.09.04-17.05.04-17.03.02) and artificial aggregate (CER 10.02.02) deriving from the recovery cycle of steel mill slag for the production of cement mixes for the foundation layers.
(2) Artificial grit deriving from the recovery cycle of steel mill slag (CER 10.02.02) for the production of bituminous conglomerates for the construction of high load road pavements. Steel mill slag recovery materials are waste from the smelting of ferrous metal alloys in second smelting cast iron and steel foundries, ferroalloy production and the steel industry.

The treatment of these materials is carried out through mechanical and technologically interconnected phases of grinding, screening, particle size selection, separation of the metal fraction (iron removal) and unwanted fractions to obtain inert fractions with suitable and selected particle size.

The research aims are to study the possibility of using materials such as CDM and steel slags in the production of cement mixes for the foundation layers and asphalt mixes for base, binder and surface layers of the road pavement. 

## 2. Materials

In this study, four mixture were analyzed: Two cement bound mixture for the foundation layer and two asphalt mixture for the upper layer of the pavement.

### 2.1. Slag in Foundation Layer

**Figure 2:**
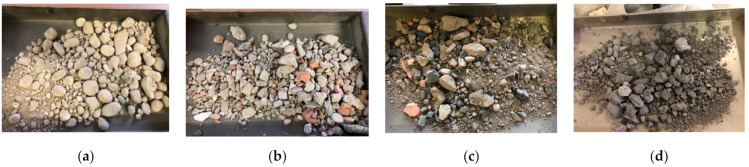
M040 virgin aggregate (**a**); M040 CDM aggregate (**b**); FM040 steel slag and CDM (**c**); oven slug (**d**).

Regarding the foundation layer, the experimental study involved the mix design and the laboratory characterization of 2 different concrete mixture. The first one, called M040, was produced with virgin aggregate coming from the cave (57%), with CDM waste aggregate (40%) and with a traditional Portland cement 32.5 (3%); the second one called FM040 was entirely produced with CDM wastes (67%) designed with steel manufacturing slag, masonry waste (30%) and with a traditional Portland cement 32.5 (3%) (Figure 2).

### 2.2. Steel Slag in Asphalt Layer

Concerning the asphalt layer, starting from a mixture of bituminous conglomerate defined as “M0” as a reference, the aim of this phase was optimizing the quantity of slag in an asphalt mixture and, subsequently, to identify for this mixture the optimum percentage of bitumen. This mixture consists mainly of natural aggregates, 10% slag content and a bitumen content of 5.5 % by weight of aggregate.

The bitumen used in the experimentation in the bituminous conglomerates part is a 50/70 bitumen with the following characteristics: penetration at 25 °C equal to 66 × 10^−1^ mm (EN1426:2015), softening point 47.6 °C (EN 1427:2015) and penetration index −1.2 (EN12591:2009).

## 3. Experimental Plan

**Figure 3:**
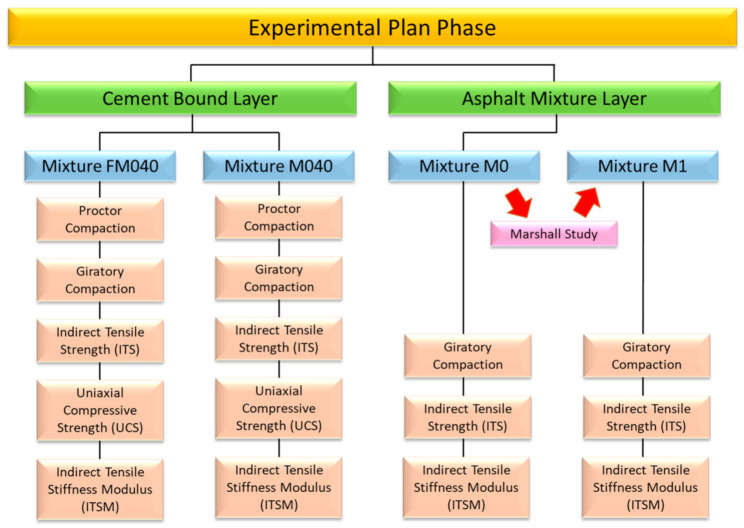
Experimental plan.

The adopted test program based on a multi-scale approach for 4 different combinations of aggregate, bitumen and concrete. Starting from the analysis of the foundation cement layers to the upper bitumen layers, following Figure 3 scheme, different laboratory tests were performed:

(1) Size distribution (EN 10343);
(2) Voids of dry compacted filler (EN 1097-5);
(3) Proctor compaction (EN 13286-2);
(4) Gyratory compaction (ASTM D6925, EN 12697/31);
(5) Marshall (EN 12697-34);
(6) Indirect tensile strength (EN 12390-6, EN 13286-42, EN 12697-23);
(7) Unconfined compressive strength (EN 13286-41);
(8) Indirect tensile stiffness modulus (EN 12697-26).

The samples analyzed for the cement bound mixture tests were different depending on the compaction mode. In particular, 3 samples were tested after proctor compaction (ITS and UCS); the FM040 mixture was tested 2 times (found similar values). Six samples were tested after gyratory compaction (ITS and ITSM).

Concerning the tests on the asphalt mixture, 3 samples per mixture were tested with the ITS test after 7 days of curing. The ITSM tests were carried out at 3 reference temperatures: 10, 20 and 30 °C. Three sample tests of each mixture for each temperature were performed.

## 4. Results

### 4.1. Cement Bound Mixture Analysis

#### 4.1.1. Size Distribution

**Figure 4:**
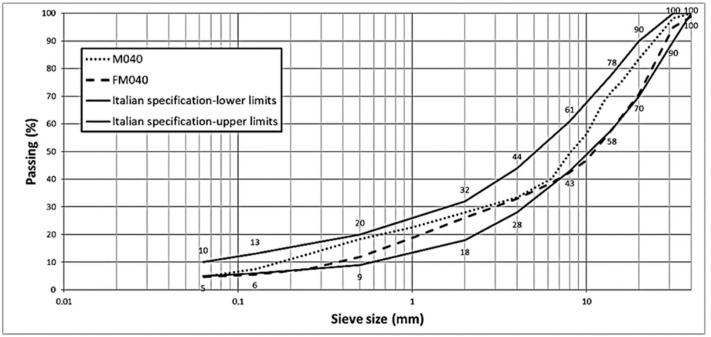
M040 and FM040 gradations.

Starting from foundation layer, the experimental mixture was designed based on the characteristics of the CDM waste, following a grain-size distribution from a typical Italian technical specification for a cement bound mixture for base layers. The gradation of the mixtures M040 and FM04 are shown in Figure 4.

The particle size distribution of aggregates depends on the properties of the mixture in terms of mix and mechanical properties. A mixture consisting predominantly of large aggregates with small percentages of finer aggregates will have low workability. Vice versa, a mixture consisting mostly of a fine fraction will have greater workability [32].

Therefore, the objective is the production of an optimal mixture following the Italian technical specification. By comparing the limits imposed by the specifications and the data obtained from laboratory tests, M040 has a greater quantity of fine fraction than mixture FM040. However, the results obtained show that both mixtures fit the limits imposed by the Italian technical specification.

#### 4.1.2. Studies of the Maximum Dry Density

A fundamental parameter for evaluating the thickening of the soil during on-site compaction is the study of how much water the mixture needs to reach the maximum dry density. Once the right amount of water to reach the maximum dry density (UNI EN 1097-5) was defined, 7 specimens were prepared for the static mechanical characterization by the means of Proctor compaction (UNI EN 13286-2, Table 1). The molds were filled with material in 5 different layers, each of which compacted by 25 blows, according to the use of a 4.5 kg hammer falling through 45.7 mm. The test procedures are all reported in the indicated standards.

The maximum dry density value(s) for the FM040 material, containing CDM wastes designed with steel manufacturing slag, is higher than M040, containing virgin aggregate coming from the cave and CDM waste aggregate. This could confirm a greater resistance of the material given by the steel slag.

#### 4.1.3. ITS and UCS Results Analysis after Proctor Compaction

After Proctor compaction (AASHTO Mod), 6 samples for M040 and 4 for FM040 were tested. The mechanical characterization was made with indirect tensile strength (ITS) and unconfined compressive strength (UCS) tests according to EN 12390-6 and UNI EN 13286-41 standards. Results of ITS are given in Table 2, while Table 3 show the UCS average result after 7 days of maturation. 

The values of ITS and UCS for the material FM040, containing CDM wastes designed with steel manufacturing slag and discarded masonry bricks are higher than M040, containing virgin aggregate coming from the cave and CDM waste aggregate, resulting in line with the specifications required by the main specifications. It is underlined that the performance is maintained even after 7 days of maturation.

#### 4.1.4. ITS Results Analysis after Gyratory Compaction

During this step, 12 specimens were compacted using gyratory compaction (ASTM D6925). According to the technical specification taken as reference, the settings for the gyratory shear compactor were: 180 gyrations, 600 kPa compaction pressure and 1.25° angle. The mechanical properties of strength were assessed by the means of the ITS tests after 7 days of curing (EN 13286-42). ITS results are given in Table 4.

After 7 days of curing, the experimental mixtures recycled show a remarkable resistance to indirect traction. FM040 sample results are better than M040. In addition, the increase in dry density (2.573 for FM040, 2.198 for M040, Table 1 and Table 2) for gyratory compaction samples, improves the mechanical performance of the mixtures compared to the proctor compacted ones.

#### 4.1.5. Indirect Tensile Stiffness Modulus Results

The mechanical analysis was completed with the dynamic characterization. The stiffness modulus was calculated according to the EN 12697-26 standard (indirect tensile configuration). 6 samples were prepared for material with gyratory compaction (same configuration previously described). The samples were tested after 7 days of curing, according to the dimensions required by the regulations. The results are shown in Table 5.

There is a consistent difference in stiffness between the two mixtures. After 7 days of curing, FM040 has a higher stiffness modulus (16,439 MPa) even if compared to the reference virgin mixture (12,430 MPa).

### 4.2. Asphalt Mixture Analysis

#### 4.2.1. Size Distribution

**Figure 5:**
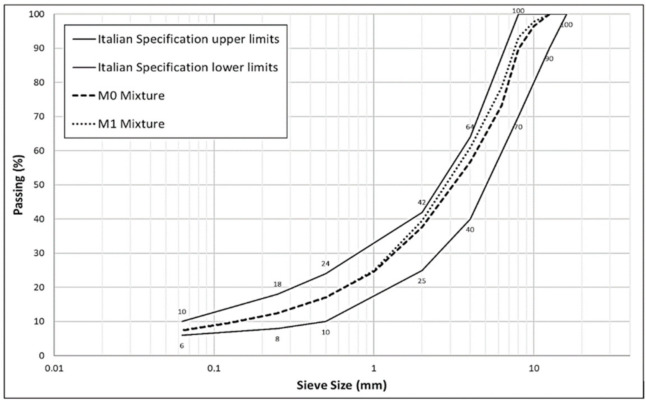
Asphalt mixtures gradation.

Based on particle size analysis, the reference curve M0 was constructed. In particular, the percentages by weight of the individual particle size fractions contained in each range were identified. The granulometric curve for mixture M0 was created in respect of a typical Italian standard. M1 was defined to increase the percentage of slag within the aggregate mixture. This mixture contains the 30% slag in the aggregate as described Figure 5.

#### 4.2.2. Marshall Analysis

**Figure 6:**
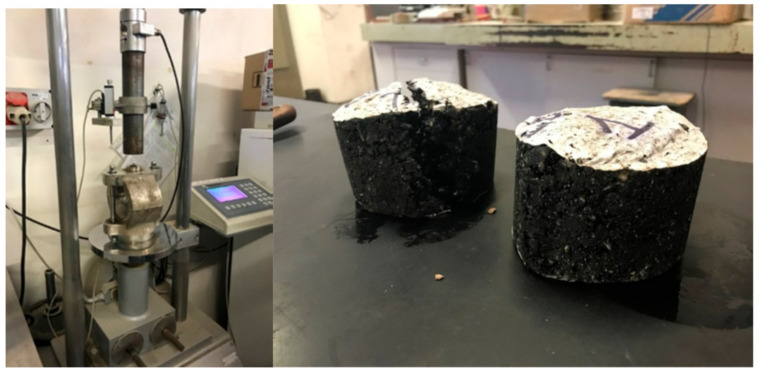
Marshall test.

To define the optimum percentage of bitumen, a total of 12 samples were prepared for the Marshall compaction study phase (EN 12697-34). Different percentages of bitumen were tested: 3 samples for each value of percentage of bitumen (3, 4, 5, 6%). Marshall tests were carried out on these specimens. The volumetric analysis was based on the evaluation of the air voids content on samples prepared with compaction energies of 75 blows on each side. Stability and displacement were extrapolated for each test pieces by calculating the average of 3 samples for each bitumen percentage (Table 6). Figure 6 shows the test configuration and, for example, two samples at the end of the test.

It is evident that, for all mixtures, the stability increases with the decrease in the percentage of bitumen and vice versa the displacements decrease with the same trend. The curve of Marshall stability thus obtained does not have the classic parabolic shape.

**Figure 7:**
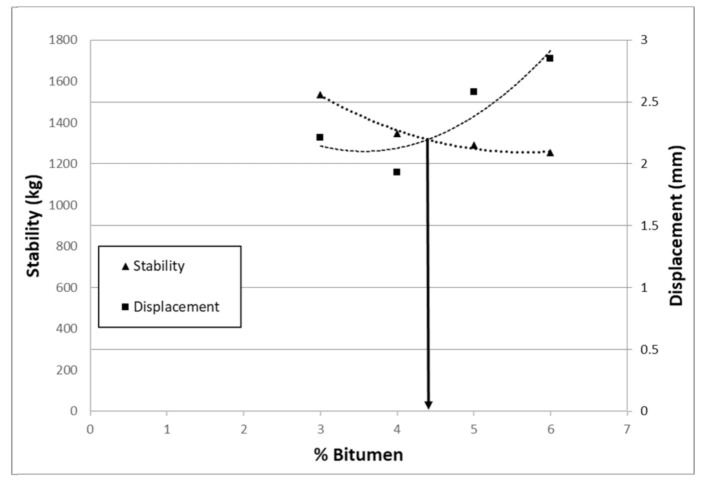
Choice of optimal bitumen percentage after marshal test.

The aggregate skeleton containing 30% slag significantly increases the stiffness of the samples and reacts mainly by reduced percentages of bitumen. The choice of optimal bitumen was therefore made by combining the results of the Marshall tests with the internal void content of each sample. The content of internal voids increases in proportion to the reduction in the percentage of bitumen (Figure 7). Considering these results, 4.5% by weight was chosen as the optimum bitumen content. This percentage represents an optimal compromise between the stability/sliding values and the percentage of internal voids.

With the optimal bitumen percentage (4.5%) obtained from the mix design, 3 other samples were produced and tested later with the Marshall test. M1 mixture containing 30% of slag in weight of aggregate is characterized by higher stability values than the mixture M0 (Table 7).

In mixture M1 (4.5% bitumen and 30% slag) the percentage of internal voids is about 8.3%, significantly higher than the 4.9% percentage of voids in mixture M0. Analyzing the ratio between stability and displacement, the M1 mixture is fully comparable to the mixture M0 in terms of mechanical performance.

#### 4.2.3. Gyratory Compaction Study

**Figure 8:**
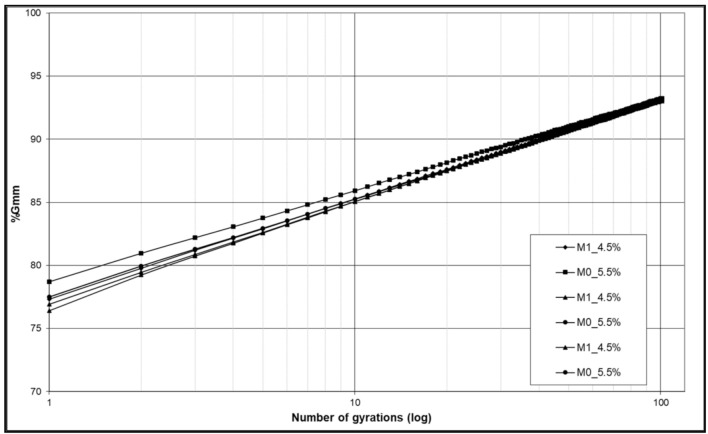
Gyratory compaction curves.

For each sample, maximum density, bulk density and void index have been determined (EN 12697/8). According to the technical specification taken as reference, the settings for the gyratory shear compactor were: 180 gyrations, 600 kPa of compaction pressure and an external angle of 1.25°. During this step, other 6 specimens (3 samples M0, 3 sample M1) were compacted with gyratory compaction (EN 12697/31). During the compaction cycles, for each sample, the mean height of the material compressed into the mold was obtained as a function of the revolutions number. The appropriate reworking of the data allowed the construction of the gyratory compaction curves and the control of their volumetric characteristics (Figure 8).

The mixture M0 characterizes a sample containing 5.5% bitumen has +1% bitumen compared to the experimental mixture containing 30% slag (mixture M1). From the thickening curve comparisons, it is possible to note that these curves have a parallel trend, and this means that the degree of thickening is the same for both types tested. 

#### 4.2.4. ITS results Analysis after Gyratory Compaction

Once again, the mechanical properties of strength were assessed by the means of the ITS (EN 12697-23) test after 7 days of curing. 

Table 8 shows the average results of indirect tensile strength tests for test and reference asphalt mixtures. In particular, the values of the tests carried out at 25 °C are shown.

First of all, it should be noted that all mixtures have values of indirect tensile strength higher than those required by the most common specifications. In relative terms, a comparison between the mixtures M0 and M1 with 30% slag shows that M1 has lower indirect traction values than the reference ones. This result, although not evident, is to be attributed to the different quantity of bitumen present in the two mixtures. In particular, the presence of 5.5% of bitumen on the mixture “M0” ensures that there is greater cohesion between the aggregates and consequently greater resistance. However, the analysis of the flow and therefore the value of CTI confirms what is seen in the previous paragraph about the increase in stiffness given by the slag to the mixture, with consequent reduction of deformability.

#### 4.2.5. Indirect Tensile Stiffness Modulus Analysis

**Figure 9:**
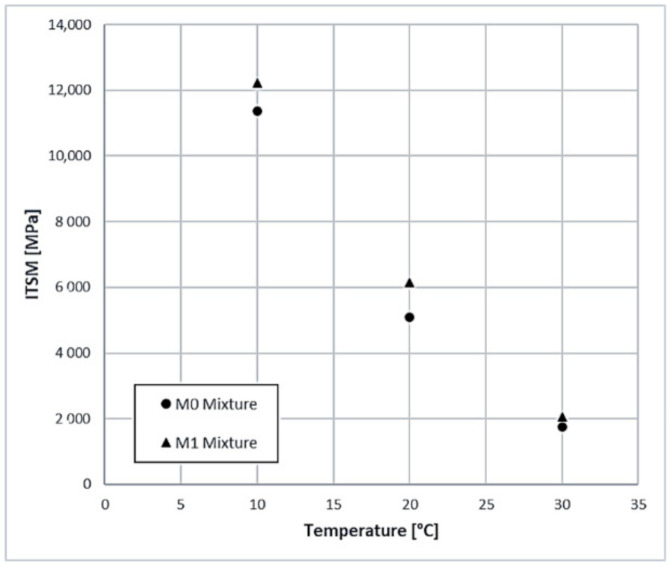
Indirect tensile resilient modulus (ITSM) average value at different temperature (10°, 20°, 30°).

The dynamic mechanical characterization of the mixtures is based on ITSM tests (EN 12697-26, part C) carried out at 3 reference temperatures: 10, 20 and 30 °C. In this way, it was possible to define the stiffness characteristics of the mixtures and verify their thermo-sensitivity. For each mixture, 3 test samples were produced with a gyratory press (UNI EN 12697), conditioned for at least 4 h at reference temperatures before testing. The average results are given in Table 9 and Figure 9.

The ITSM test provides guidance on the stiffness characteristics of mixtures. All M1 mixtures show ITSM values higher than the respective bituminous comparison conglomerates. Even in this case, a higher percentage of slag leads to an overall improvement of the mechanical characteristics in the experimental bituminous mixtures. In absolute terms, the stiffening of the mixtures is visible throughout the temperature range. Samples with a stiffer modulus are those containing 4.5% bitumen and 30% slag.

The two mixtures had the same bitumen and degree of thickening. The increase in stiffness is due to the lithic skeleton of the mixture and consequently to the greater quantity of slag which intervenes on the bearing capacity of the pavement.

## 5. Conclusions

The aim of the present work was to evaluate the physical and mechanical properties of a cement bound and asphalt mixture made with different percentage of CDM materials and steel slag. The experimental program was divided according to the experimental plan in Figure 3. Starting from the analysis of the foundation cement layers to the upper bitumen layers, different laboratory tests were performed. According to the experimental results presented in this study, the following conclusion can be drawn for the laboratory phase:

(1) Regarding the cement bound mixture, static mechanical characterization tests (ITS, UCS) highlight that results are not far from those obtained with the virgin mixture and satisfy the principal requirement imposed by the technical specifications. Additionally, the ITSM results confirm what is found in the literature: there is a consistent difference in stiffness between the mixture analyzed. The mixture containing steel slag has a higher stiffness modulus even if compared to the reference virgin mixture;
(2) The asphalt mixture that contains a higher percentage of slugs in weight of aggregate is characterized by higher stability and stiffness. The optimum mixture was obtained using 4.5% bitumen and 30% slag.

Research has shown the possibility of using these materials in road infrastructures in order to promote the objectives set out by the sustainable development goals to promote the circular economy and sustainable construction.

## Figures and Tables

**Table 1:** UNI EN 13286-2 results for M040.

Mixture	Water Content during Compaction (%)	Wet Density (g/cm^3^)	Dry Density (g/cm^3^)	Optimum Water Content (%)	Max Dry Density (g/cm^3^)
M040	5.0	2.276	2.167	6.0	2.198
M040	5.9	2.278	2.151		
M040	6.7	2.332	2.185		
FM040	1.5	2.419	2.384	5.6	2.573
FM040	3.6	2.549	2.460		
FM040	8.2	2.703	2.498		
FM040	4.0	2.709	2.604		

**Table 2:** Indirect tensile strength (ITS) results.

Name	Height (mm)	Ø (mm)	Load (daN)	Displacement (mm)	Rt (MPa)	Average (MPa)
M040	177.8	150	1665	0.46	0.40	0.32
M040	177.8	150	920	0.94	0.22	
M040	177.8	150	1380	0.74	0.33	
FM040	177.8	150	2500	1.1	0.60	0.57
FM040	177.8	150	2285	0.74	0.55	

**Table 3:** Unconfined compressive strength (UCS) results.

Name	Height (mm)	Ø (mm)	Load (daN)	Rc (MPa)	Average (MPa)
M040	177.8	150	5530	3.13	2.90
M040	177.8	150	3055	1.73	
M040	177.8	150	6765	3.83	
FM040	177.8	150	6245	3.53	3.84
FM040	177.8	150	7340	4.15	

**Table 4:** Indirect tensile strength (ITS) results.

Name	Height (mm)	Ø (mm)	Load (daN)	Displacement (mm)	Rt (MPa)	Average (MPa)
M040_1	57.03	150	790	0.52	0.59	0.49
M040_2	63.39	150	745	0.54	0.50	
M040_3	64.2	150	660	0.44	0.44	
M040_4	59.21	150	600	0.48	0.43	
M040_5	62.73	150	680	0.56	0.46	
M040_6	58.57	150	725	0.54	0.53	
FM040_1	53.29	150	780	0.30	0.62	0.65
FM040_2	48.75	150	740	0.48	0.64	
FM040_3	48.68	150	600	0.42	0.52	
FM040_4	51.87	150	845	0.52	0.69	
FM040_5	49.17	150	680	0.38	0.59	
FM040_6	52.72	150	1000	0.58	0.81	

**Table 5:** Indirect tensile stiffness modulus results.

Mixture	Average Height (mm)	Temperature (°C)	D1 (MPa)	D2 (MPa)	Average (MPa)	Total (MPa)
M040_1	62.73	19	15,895	15,620	15,757.5	12,430
M040_2	58.57	19	7642	7652	7647	
M040_3	57.033	18	14,741	15,155	14,948	
M040_4	63.39	18	18,551	20,391	19,471	
M040_5	64.2	19	8173	8034	8103.5	
M040_6	59.21	19	8847	8462	8654.5	
FM040_1	52.29	18	20,438	17,009	18,723.5	16,440
FM040_2	48.75	18	14,131	13,543	13,837	
FM040_3	48.68	17	14,083	13,051	13,567	
FM040_4	51.87	17	15,898	15,877	15,887.5	
FM040_5	49.17	17	16,976	17,135	17,055.5	
FM040_6	52.726	17	18,983	20,155	19,569	

**Table 6:** Marshall test results.

Bitumen (%)	Stability (kg)	Displacement (mm)	Stiffness (kg/mm)	Weight/Volume (g/cm^3^)	Void (%)
**6**	1255.00	2.85	445.86	2.62	3.82
**5**	1289.00	2.58	499.30	2.60	6.40
**4**	1346.33	1.93	697.42	2.59	7.99
**3**	1536.00	2.21	709.36	2.54	11.40

**Table 7:** Comparison table after Marshall study phase.

Mixture	Bitumen (%)	Stability (kg)	Displacement (mm)	Stiffness (kg/mm)	Weight/Volume (g/cm^3^)	Void (%)
**M0**	5.5	1334.00	2.28	585.46	2.48	4.88
**M1**	4.5	1391.00	2.38	537.39	2.56	8.31

**Table 8:** ITS test results.

Name	ITS (MPa)	CTI (MPa)	Average ITS (MPa)
M0	0.90	70.90	0.94
M0	0.95	79.03	
M0	0.97	81.32	
M1	0.92	81.26	0.92
M1	0.87	77.38	
M1	0.98	92.46	

**Table 9:** Average results of ITSM test at 10, 20 and 30 °C for M0.

Sample	ITSM (MPa) @ 10 °C	ITSM (MPa) @ 20 °C	ITSM (MPa) @ 30 °C
M0	11641	4573	1575
	11251	5470	1841
	11232	5261	1810
M1	11129	5165	1980
	12616	6263	1902
	13907	7017	2200

## Data Availability

Data sharing is not applicable to this article.
